# Infants Hospitalized with Lower Respiratory Tract Infections Were More Likely to Develop Asthma

**DOI:** 10.3390/arm90040034

**Published:** 2022-07-21

**Authors:** Masafumi Zaitsu, Shun Morita

**Affiliations:** National Hospital Organization Ureshino Medical Center, 4279-3 Shimojuku Kou, Ureshino 8430393, Japan; shun.morita81@gmail.com

**Keywords:** bronchial asthma, infant, lower respiratory tract infection, wheezing

## Abstract

**Highlights:**

- Lower respiratory tract infections in infancy may be involved in the development of asthma.
- The severity of lower respiratory tract infections in hospitalized infants but not the particular viral pathogen causing the infection may be associated with later asthma onset.
- The modulation of the immune response to lower respiratory tract infections in infancy might be a therapeutic target for the prevention of asthma and/or recurrent wheezing.

**Abstract:**

Introduction: Lower respiratory tract infections (LRTIs) have been reported to possibly initiate the development of asthma in children. However, the role of LRTIs in infantile asthma remains controversial. The goal of this study is to investigate whether LRTIs in hospitalized infants are involved in the development of asthma. Materials and Methods: The subjects were 251 infants under 2 years of age who were admitted to our hospital with an RTI (59 cases of upper RTI (URTIs) with upper respiratory tract inflammation and pharyngeal tonsillitis; 192 cases of LRTIs with bronchitis, pneumonia, and bronchiolitis). Pathogens of viral infections were examined at admission using viral antigen test kits that could be used in ordinary clinical practice in Japan. When the children reached the age of 3 years, a survey was conducted by mailing a questionnaire to determine the symptoms, diagnosis, and treatment of asthma. Results: The mailed questionnaires were returned by 116 of the 251 subjects. On the questionnaire, the diagnosis of asthma and treatment for asthma were significantly higher in hospitalized infants with LRTIs than in those with URTIs. By diagnosis of LRTIs, infants with pneumonia and bronchiolitis were significantly more likely to develop asthma. However, on pathogen-specific examination, there was no difference in the development of asthma among infants with LRTIs. Conclusion: LRTI in infancy may be involved in the development of asthma. The severity of LRTI in hospitalized infants, but not the particular viral pathogen causing infection, may be associated with later asthma onset.

## 1. Introduction

Lower respiratory tract infections (LRTIs), including pneumonia and bronchiolitis, in early life could lead to airway obstruction and hyper-reactivity and the development of asthma [1,2,3,4,5,6]. The most important pathogen of LRTIs is considered to be viral infection [1,3]. Respiratory syncytial virus (RSV), rhinovirus (RV), and human metapneumovirus (hMPV) have been reported to be associated with the development of asthma [1,3,6,7,8,9]. However, the mechanisms are not fully understood, and whether the severity of LRTI is associated with the development of asthma in infants and whether the particular viral pathogen initiates the onset of asthma also remain controversial [10,11,12].

Thus, a questionnaire survey at the age of 3 years was conducted to evaluate whether infants under 2 years of age hospitalized with LRTIs developed asthma more frequently than those with upper RTIs (URTIs). Furthermore, the relationship between LRTIs due to specific viral infections and the onset of asthma was investigated using virus antigen test kits. 

## 2. Materials and Methods

From April 2016 through March 2017, a total of 251 infants, younger than 2 years (range 0–23 month) of age in the Saga area of Western Kyushu Island, Japan, who were admitted to the National Hospital Organization Ureshino Medical Center with a diagnosis of respiratory infection (59 cases of URTI, 192 cases of LRTIs) were enrolled. URTIs, bronchiolitis, and pneumonia were diagnosed according to the following diagnostic criteria. URTIs were diagnosed in patients who had a sore throat, nasal discharge, and nasal obstruction as the main symptoms. Bronchitis was diagnosed in patients who had airway symptoms such as fever, cough, and sputum, but did not have clear abnormal shadows on chest radiographs. Bronchiolitis was diagnosed in patients who had symptoms such as wheezing, tachypnea, and depressed breathing following nasal discharge and nasal obstruction, and showed enlarged lung volume and increased permeability on chest radiographs. Pneumonia was diagnosed in patients who had airway symptoms such as fever and cough and acute new infiltration shadows on chest radiographs. Infants born prematurely or who had severe congenital diseases, such as a congenital heart disease, an airway anomaly, or a metabolic disease, were excluded. The infants who had been diagnosed as having an allergic disease by a physician and had repeated wheezing episodes before hospitalization were also excluded. Infants who were hospitalized multiple times before this hospitalization and who had been diagnosed with bronchial asthma at the time of admission or in the past were also excluded. Informed consent was obtained from the parents of all patients. At the first hospitalization, age, sex, family history of allergic disease, including atopic symptoms of parents and sibling, and laboratory findings such as the number of white blood cells (WBCs), C-reactive protein (CRP) level, and immunoglobulin E (IgE) level were checked in these subjects. Pathogens were examined using antigen test kits that could be used in ordinary clinical practice in Japan. RSV infection, hMPV infection, adenovirus (ADV) infection, influenza (Flu) infection, and streptococcus infection were confirmed using kits (Check RSV, Check hMPV, and Check Ad; Meiji Seika Pharma Co., Ltd., Tokyo, Japan; Rapidtesta-Flu and Rapidtesta-Strep A, Sekisui Medical Co., Ltd., Tokyo, Japan) for identification of antigens in nasopharyngeal secretions. Bacterial culture of nasopharyngeal swabs was also performed. No patients with positive results for *Chlamydia trachomatis* by a specific ELISA kit (HITAZYME CHLAMYDIA TRACHOMATIS, Hitachi Chemical Co., Ltd., Tokyo, Japan) or *Chlamydia psittaci* by a complement fixation (CF) kit (CHLAMYDIA PSITTACI-CF test, Denkaseiken Co., Ltd., Tokyo, Japan), *Mycoplasma pneumoniae* by an indirect microparticle agglutinin method kit (Serodia-MYCO II, Fujirebio Co., Ltd., Tokyo, Japan), and *Pertussis* by an agglutinin method kit (*Pertussis*-agglutinin test, Denkaseiken Co., Ltd., Tokyo, Japan) were confirmed in this study.

Next, when the patients reached the age of 3 years, a questionnaire survey was conducted by mailing a questionnaire to determine the symptoms (Q1), the diagnosis (Q2), and the treatment (Q3) of asthma, as shown in Table 1. The questions (Q1, Q2, and Q3) in the questionnaire were about whether the diagnostic criteria for asthma of the guidelines were met (Answers A 1 to A 3 do not meet the diagnostic criteria for infant asthma, and A 4 and A 5 meet the diagnostic criteria for infant asthma), using the Japanese Pediatric Guidelines for the Treatment and Management of Asthma in Japan 2017 [13,14]. Whether the infants hospitalized with LRTIs developed asthma more frequently than those with URTIs was evaluated overall, by diagnosis, and by pathogenic virus in hospitalized infants with LRTIs.

The study protocol was approved by the clinical research ethics review board at Ureshino Medical Center (approval number: 16-01).

### Statistical Analysis

The results were analyzed using the chi-squared test for categorical variables. The Mann-Whitney U test was used for continuous variables. A *p* value of less than 0.05 was considered significant.

## 3. Results

The mailed questionnaire was returned by 116 of the 251 subjects (87/192 with LRTI, 29/59 with URTI). Table 2 shows the diagnosis and pathogens detected of 116 subjects. The following diagnoses were confirmed: 31 bronchitis (35.6%), 41 pneumonia (47.1%), and 15 bronchiolitis (17.2%) in the 87 patients with LRTIs; and 14 upper respiratory tract inflammation (48.3%) and 15 pharyngeal tonsillitis (51.7%) in the 29 patients with URTIs. The following pathogens were detected: 37 RSV (42.5%), 14 hMPV (16.1%), 2 Flu A (2.3%), 2 ADV (2.3%), and 15 “Undetected” (17.2%) in the 87 patients with LRTIs; additionally, 3 Flu A (10.3%), 3 ADV (10.3%), 2 RSV (6.9%), and 21 “Undetected” (72.4%) in the 29 patients with URTIs. Characteristics and questionnaire results of the 116 subjects are shown in Table 3. Sex, age at admission, age at questionnaire, family history of allergic disease, WBC count, serum CRP level, and serum IgE level did not differ significantly between infants with URTIs and those with LRTIs. The duration of hospitalization was significantly longer in infants with LRTIs than in those with URTIs. This may indicate that more severe infections result in longer hospital stays. The results of the questionnaire regarding the development of asthma showed that the frequencies of a diagnosis of asthma (Q2) and of treatment prescription for asthma (Q3) were significantly higher in hospitalized infants with LRTIs (28/87 (32.2%), odds ratio 6.4, 95%CI 1.4–28.9; and 43/87l (49.4%), odds ratio 2.6, 95%CI 1.0–6.4) than in those with URTIs (2/29 (6.9%) and 8/29 (27.6%)). This result suggested that LRTIs might be involved in the development of asthma. There was no significant difference in the question on asthma symptoms (Q1). Cough and wheezing were common symptoms in infants with RTIs and may not have been significantly different, since they were also observed as symptoms in infants with RTIs who did not develop asthma.

The characteristics and questionnaire results of infants with LRTIs by diagnosis are shown in Table 4. Sex, age at the questionnaire, family history of allergic disease, WBC count, and serum IgE levels did not differ significantly among infants with bronchitis, pneumonia, and bronchiolitis. The age at admission was significantly higher in infants with pneumonia than in infants with bronchitis, and that in infants with bronchiolitis was significantly lower than that in infant with bronchitis. The duration of hospitalization was significantly longer in infants with pneumonia and bronchiolitis than in infants with bronchitis. The questionnaire results by diagnosis in hospitalized infants with LRTI showed that the infants with pneumonia (17/41 (41.5%), OR 4.8, 95%CI 1.4–16.2) and bronchiolitis (7/15 (46.7%), OR 5.9, 95%CI 1.4–25.4) were significantly more likely to be diagnosed with asthma (Q2) than those with bronchitis (4/31 (12.9%)) (Table 4).

The characteristics of the subjects and questionnaire results by pathogen are shown in Table 5. The data were compared with RSV, which was considered to be an important cause of LRTI in infants. Sex, age at the questionnaire, family history of allergic disease, WBC count, and serum IgE level did not significantly differ among infants with RSV, hMPV, and “Undetected” infections. The age at admission was significantly higher in infants with hMPV and “Undetected” infections than in infants with RSV. As is well known, RSV infection occurred in younger infants. However, the analysis of the questionnaire results by pathogen showed that the development of asthma (symptoms (Q1), diagnosis (Q2), and prescription (Q3)) did not differ among the groups with RSV, hMPV, and “Undetected” infections in hospitalized infants with LRTIs. 

## 4. Discussion

The present study shows that hospitalized infants with LRTIs are significantly more likely to develop asthma at 3 years of age, and the reason for developing asthma in hospitalized infants with LRTI was not the type of viral pathogen, but the difference in diagnosis, probably the difference in LRTI severity. 

LRTIs, especially due to viral infection, are common causes of asthma exacerbations in children [2,6,7,8,9,15,16]. In addition, several reports of evidence suggest that viral infection in early childhood is associated with the development of asthma [1,3,6,9,12].RSV is a common cause of LRTIs in infants and is also a well-known cause of wheezing, and infants with RSV-induced bronchiolitis frequently develop asthma [2,17]. Sigurs et al. followed hospitalized infants with bronchiolitis under the age of 1 year up to the age of 18 years, and they reported that such infants were diagnosed with asthma at a significantly higher rate than controls [1]. The infants with bronchiolitis also had a high rate of allergic sensitization [18]. Stein et al. showed that LRTI with RSV in infants younger than 3 years was a risk factor for the subsequent development of wheezing up to 11 years of age, but not at age 13 years, and the rate of allergic sensitization in these infants was not different from that of controls [3]. In addition, it was considered that LRTI with RV was involved not only in the exacerbation of asthma, but also in the onset of asthma [6]. Rubner et al. concluded in a prospective cohort study that the persistence of asthma at age 13 years was associated with RV-induced LRTI during the first 3 years of life [19]. Liu et al. showed, in their meta-analysis, that LRTI caused by RV in the first 3 years may be associated with the subsequent development of asthma [20]. It has been reported that hMPV infection also plays a role in initiating asthma development in children [7]. Garcia-Garcia et al. demonstrated that children aged 3–5 years and hospitalized for hMPV-bronchiolitis were more likely to develop asthma in the preschool years [9]. As shown in previous reports, the present study also showed that LRTIs, mostly caused by viral infections, play a role in the diagnosis of asthma at age 3 years. In our view, the mechanism remains unclear, but LRTI due to viral infection appears to be an important risk factor for asthma development.

However, a recent systematic review has reported that the contribution of viral infection, especially RSV, to asthma development may not be causal [21,22]. Several studies have previously shown that the severity and frequency of LRTIs are associated with asthma, rather than a particular viral infection. Rolfsjord et al. showed that the severity of acute bronchiolitis in infants was associated with reduced quality of life later due to frequent wheezing [10]. Mansbach et al. demonstrated, in a multicenter cohort study, that infants who underwent intensive care during hospitalization with bronchiolitis had a significantly higher risk of recurrent wheeze at age 3 years, and the severity of bronchiolitis was related to asthma development in a prospective study [11]. Hunderi et al. showed that recurrent wheeze at 2 years of age was not significantly associated with specific viruses during acute bronchiolitis leading to hospitalization in the first year, and they concluded that acute bronchiolitis by specific viruses in infancy did not increase the risk of asthma by 2 years of age [23]. In the present study, the development of asthma in infants with LRTIs was not different depending on the type of virus pathogen, but it was significantly different depending on the diagnosis of LRTI. The duration of hospitalization was significantly longer in infants with pneumonia and bronchiolitis than in those with bronchitis. This may indicate that more severe and/or prolonged symptoms of LTRIs, such as pneumonia and bronchiolitis, result in longer hospital stays. The questionnaire results by diagnosis in infants with LRTI showed that the infants with pneumonia and bronchiolitis were significantly more likely to develop asthma. The analysis of the questionnaire results by pathogen showed that the development of asthma did not differ between groups of pathogens. Then, our results may show that severe and/or prolonged LRTI that required hospitalization in infancy, such as pneumonia and bronchiolitis, but not the particular viral pathogen, is associated with later asthma development. Furthermore, Bønnelykke et al. reported that the number of respiratory infection episodes in the first years of life, but not the particular viral infection, was associated with later asthma development [12]. De Oliveira et al. also demonstrated that recurrent LRTIs in childhood were associated with the presence of asthma [24]. They thought that the frequency of LRTIs in early infancy is a risk factor for recurrent wheezing, rather than the type of virus. Nathan et al. reported that infants admitted for LRTIs had frequent respiratory sequelae, of which preschool wheeze was common [25]. We also consider that the development of asthma may depend on the severity and the frequency of LRTIs and the degree of respiratory damage and sequelae in infants who required hospitalization with LRTIs, regardless of the particular viral infection. Abdullah et al. suggested that the contribution of LRTIs with viral infections to the development of asthma is associated with viral injury, disease severity, pre-existing abnormal lung function, genetic susceptibility, and environmental factors [22]. Several additional factors may be associated with the development of asthma after LRTIs with viral infections.

Our study has several limitations. In our study, RV could not be tested since it cannot be used in normal clinical practice in Japan. The patients with RV infection are probably included in the “undetected” group in our study. There was no difference between pathogens associated with the development of asthma in the “undetected” group, which is thought to contain RV. However, RV is considered to be an important cause of the onset and exacerbation of asthma and requires further investigation involving the detection of RV. Another limitation is about the diagnostic criteria for asthma. When the patients reached the age of 3 years, we conducted a questionnaire survey to find out whether the patient was diagnosed with asthma. At the age of 3 years, it is difficult to accurately diagnose asthma based on the information in the questionnaire.

The present study confirmed that infants hospitalized with LRTIs were more likely to develop asthma later. The severity of LRTIs in hospitalized infants, but not the particular viral pathogen, may be associated with later asthma development. The modulation of the LRTI-immune response in infancy might be a therapeutic target for the prevention of asthma and/or recurrent wheezing. Further large-scale studies are needed to confirm the relationship between LRTIs and asthma development in early childhood.

## Figures and Tables

**Table 1:** Questionnaire questions.

Choose an Answer from Five Options for Each Question.
Q1: Have you ever had a cough or wheezing (beeping breath sounds) after discharge? A1: Not at all. A2: Almost none. A3: Occasionally (Once or twice a year) A4: Sometimes (once a month, or more than 3 times in total after discharge.) A5: Common (more than twice a month) Q2: Have you ever been diagnosed or suspected of having bronchial asthma at a medical institution (hospital, clinic, etc.) after discharge? A1: Not at all. A2: Almost none. Doctor said, “prone to coughing”. A3: Occasionally. Although not diagnosed with asthma, doctor said, “weak bronchi”. A4: Doctor said, “maybe or suspected asthma”, or “You have a lot of coughing and wheezing.” A5: Doctor said, “asthma (diagnosed asthma)”. Q3: Have you ever been prescribed a medicine for bronchial asthma (leukotriene receptor antagonists, beta agonists, and inhaled corticosteroids, etc.) at a medical institution after discharge? A1: Not at all. A2: Almost none. A3: Occasionally (Only when coughing or wheezing occurs once or twice a year) A4: Sometimes (once a month, or more than 3 times in total after discharge.) A5: Common (more than twice a month, or prescribed on a regular basis)

**Table 2:** Diagnoses and detected pathogens in hospitalized infants with respiratory tract infections.

	URTIs	LRTIs
	(*n* = 29)	(*n* = 87)
Diagnosis (*n*)	Upper respiratory tract inflammation 14	Bronchitis 31
	Pharyngeal tonsillitis 15	Pneumonia 41
		Bronchiolitis 15
Pathogen (*n*)	Flu A 3	RSV 37
	ADV 3	hMPV 14
	RSV 2	Flu A 2
	Undetected 21	ADV 2
		Undetected 32

URTIs: upper respiratory tract infections; LRTIs: lower respiratory tract infections; Flu A: influenza virus type A; ADV: adenovirus; RSV: respiratory syncytial virus; hMPV: human metapneumovirus.

**Table 3:** Characteristics of the subjects and questionnaire results of hospitalized infants with respiratory tract infections.

	URTIs	LRTIs
	(*n* = 29)	(*n* = 87)
Sex (M/F) (n)	19/10	55/32
Age at questionnaire (months)	36 (36–37)	36 (36–37)
Age at admission (months)	6 (0–23)	7 (0–23)
Family history (*n*)	7	22
WBC (/µL)	11,600 (5200–33,300)	11,100 (1700–32,500)
IgE (IU/mL)	43.8 (12.0–206.9)	39.3 (12.0–320.1)
CRP (mg/dL)	1.70 (0.05–13.08)	1.01 (0.01–15.27)
Hospitalization (days)	5 (2–11)	6 (3–14) *
Results of questionnaire		
Q1 Symptoms of asthma (*n*), (%)	6 (20.7%)	28 (32.2%)
Q2 Diagnosis of asthma (*n*), (%)	2 (6.9%)	28 (32.2%) ^OR 6.4 (95%CI 1.4–28.9)^ *
Q3 Prescription of asthma (*n*), (%)	8 (27.6%)	43 (49.4%) ^OR 2.6 (95%CI 1.0–6.4)^ *

URTIs: upper respiratory tract infections; LRTIs: lower respiratory tract infections; Family history: family history of allergic disease; WBC: white blood cell’ IgE: immunoglobulin E; CRP: C-reactive protein; OR: odds ratio; 95%CI: 95% confidence interval. Data are presented as the No. or medians (range). * *p* < 0.05 vs. URTIs.

**Table 4:** Characteristics and questionnaire results of hospitalized infants with lower respiratory tract infections by diagnosis.

	Bronchitis	Pneumonia	Bronchiolitis
	(*n* =31)	(*n* = 41)	(*n* = 15)
Sex (M/F) (*n*)	21/10	26/15	8/7
Age at questionnaire (months)	36 (36–37)	36 (36–37)	36 (36–37)
Age at admission (months)	5 (0–17)	11(0–23) *	2 (0–7) *
Family history (*n*)	7	11	4
WBC (/µL)	11,100 (1700–20,300)	11,300 (4100–32,500)	10,000 (5000–14,300)
IgE (IU/mL)	37.5 (12.0–320.1)	42.5 (12.0–285.4)	38.4 (12.0–305.4)
CRP (mg/dL)	1.15 (0.01–6.8)	1.62 (0.03–15.27) *	0.33 (0.01–7.41) *
Hospitalization (days)	5 (3–12)	6 (3–14) *	6 (5–14) *
Results of questionnaire			
Q1 Symptoms of asthma (*n*)	6	16	6
Q2 Diagnosis of asthma (*n*)	4	17 ^OR 4.8 (95%CI 1.4–16.2)^ *	7 ^OR 5.9 (95%CI 1.4–25.4)^ *
Q3 Prescription of asthma (*n*)	13	21	9

Family history: family history of allergic disease; WBC: white blood cell; IgE: immunoglobulin E; CRP: C–reactive protein; OR: odds ratio; 95%CI: 95% confidence interval. Data are presented as the No. or medians (range). * *p* < 0.05 vs. bronchitis.

**Table 5:** Characteristics and questionnaire results of hospitalized infants with lower respiratory tract infections by pathogen.

	RSV	hMPV	Undetected
	(*n* =37)	(*n* = 14)	(*n* = 32)
Sex (M/F) (*n*)	23/14	8/6	20/12
Age at questionnaire (months)	36 (36–37)	36 (36–37)	36 (36–37)
Age at admission (months)	4 (0–21)	15(4–23) *	7 (1–22) *
Family history (*n*)	9	4	8
WBC (/µL)	10,000 (1700–32,500)	9400 (4100–17,900)	12,150 (8800–22,300)
IgE (IU/mL)	40.3(12.0–305.4)	38.2 (12.0–280.6)	37.7 (12.0–320.1)
CRP (mg/dL)	0.42 (0.01–7.41)	1.23 (0.03–10.23) *	2.15 (0.01–15.27) *
Hospitalization (days)	6 (4–14)	5.5 (3–13)	6 (3–13)
Results of questionnaire			
Q1 Symptoms of asthma (*n*)	9	5	13
Q2 Diagnosis of asthma (*n*)	9	6	13
Q3 Prescription of asthma (*n*)	15	8	18

RSV: respiratory syncytial virus; hMPV: human metapneumovirus; Family history: family history of allergic disease; WBC: white blood cell; IgE: immunoglobulin E; CRP: C-reactive protein. Data are presented as the No. or medians (range). * *p* < 0.05 vs. RSV.
